# Prof. Edward Warren's Surgical Clinics

**Published:** 1867-12

**Authors:** 


*Prof. Edward Warren's Surgical Clinics*—Prof. Warren of the Washington Medical College of this city, has extended an invitation to the Students of the Baltimore College of Dental Surgery to attend his *Surgical Clinics* during the entire session of the College. This favor from Prof. Warren is highly appreciated by both the Faculty and Students of the Baltimore College of Dental Surgery and will prove of great benefit to those interested.

